# Supplementary material for: Unveiling the Bioactive Potential of the Invasive Jellyfish Phyllorhiza punctata Through Integrative Transcriptomic and Proteomic Analyses
Source: Biomolecules. 2025 Aug 4;15(8):1121. doi: 10.3390/biom15081121 (PMC12383608; doi:10.3390/biom15081121)
Supplement: Supplementary file 1 [file biomolecules-15-01121-s001.zip › Suplementary Material/FileS3/PEPFOLD4/XP_065064539_9968/clusters.html]

 PEP-FOLD clusters


Download archive of all models  here   

|  |  |  |
| --- | --- | --- |
| # | file | sOPEP |
| # cluster 1 - Size: 19 |  |  |
|  | XP\_065064539\_9968-model1 | -12.2879 |
|  | XP\_065064539\_9968-model1.1 | -11.9283 |
|  | XP\_065064539\_9968-model1.2 | -11.8881 |
|  | XP\_065064539\_9968-model1.3 | -11.8881 |
|  | XP\_065064539\_9968-model1.4 | -11.8708 |
|  | XP\_065064539\_9968-model1.5 | -11.8458 |
|  | XP\_065064539\_9968-model1.6 | -11.8231 |
|  | XP\_065064539\_9968-model1.7 | -11.8204 |
|  | XP\_065064539\_9968-model1.8 | -11.8111 |
|  | XP\_065064539\_9968-model1.9 | -11.8103 |
|  | XP\_065064539\_9968-model1.10 | -11.7923 |
|  | XP\_065064539\_9968-model1.11 | -11.7853 |
|  | XP\_065064539\_9968-model1.12 | -11.7677 |
|  | XP\_065064539\_9968-model1.13 | -11.7604 |
|  | XP\_065064539\_9968-model1.14 | -11.7451 |
|  | XP\_065064539\_9968-model1.15 | -11.7442 |
|  | XP\_065064539\_9968-model1.16 | -11.7158 |
|  | XP\_065064539\_9968-model1.17 | -11.6304 |
|  | XP\_065064539\_9968-model1.18 | -11.5913 |
| # cluster 2 - Size: 7 |  |  |
|  | XP\_065064539\_9968-model2 | -12.1417 |
|  | XP\_065064539\_9968-model2.1 | -11.9558 |
|  | XP\_065064539\_9968-model2.2 | -11.8249 |
|  | XP\_065064539\_9968-model2.3 | -11.8183 |
|  | XP\_065064539\_9968-model2.4 | -11.7913 |
|  | XP\_065064539\_9968-model2.5 | -11.7555 |
|  | XP\_065064539\_9968-model2.6 | -11.7184 |
| # cluster 3 - Size: 31 |  |  |
|  | XP\_065064539\_9968-model3 | -12.0982 |
|  | XP\_065064539\_9968-model3.1 | -12.0944 |
|  | XP\_065064539\_9968-model3.2 | -12.0492 |
|  | XP\_065064539\_9968-model3.3 | -11.9372 |
|  | XP\_065064539\_9968-model3.4 | -11.8592 |
|  | XP\_065064539\_9968-model3.5 | -11.8306 |
|  | XP\_065064539\_9968-model3.6 | -11.8264 |
|  | XP\_065064539\_9968-model3.7 | -11.7935 |
|  | XP\_065064539\_9968-model3.8 | -11.7807 |
|  | XP\_065064539\_9968-model3.9 | -11.7634 |
|  | XP\_065064539\_9968-model3.10 | -11.7588 |
|  | XP\_065064539\_9968-model3.11 | -11.7465 |
|  | XP\_065064539\_9968-model3.12 | -11.7362 |
|  | XP\_065064539\_9968-model3.13 | -11.7357 |
|  | XP\_065064539\_9968-model3.14 | -11.7335 |
|  | XP\_065064539\_9968-model3.15 | -11.7295 |
|  | XP\_065064539\_9968-model3.16 | -11.7295 |
|  | XP\_065064539\_9968-model3.17 | -11.7246 |
|  | XP\_065064539\_9968-model3.18 | -11.7227 |
|  | XP\_065064539\_9968-model3.19 | -11.7111 |
|  | XP\_065064539\_9968-model3.20 | -11.7066 |
|  | XP\_065064539\_9968-model3.21 | -11.6944 |
|  | XP\_065064539\_9968-model3.22 | -11.6904 |
|  | XP\_065064539\_9968-model3.23 | -11.6565 |
|  | XP\_065064539\_9968-model3.24 | -11.6448 |
|  | XP\_065064539\_9968-model3.25 | -11.6418 |
|  | XP\_065064539\_9968-model3.26 | -11.6367 |
|  | XP\_065064539\_9968-model3.27 | -11.6206 |
|  | XP\_065064539\_9968-model3.28 | -11.5526 |
|  | XP\_065064539\_9968-model3.29 | -11.5279 |
|  | XP\_065064539\_9968-model3.30 | -11.4479 |
| # cluster 4 - Size: 25 |  |  |
|  | XP\_065064539\_9968-model4 | -12.0896 |
|  | XP\_065064539\_9968-model4.1 | -11.9811 |
|  | XP\_065064539\_9968-model4.2 | -11.9666 |
|  | XP\_065064539\_9968-model4.3 | -11.9665 |
|  | XP\_065064539\_9968-model4.4 | -11.9457 |
|  | XP\_065064539\_9968-model4.5 | -11.9457 |
|  | XP\_065064539\_9968-model4.6 | -11.9015 |
|  | XP\_065064539\_9968-model4.7 | -11.886 |
|  | XP\_065064539\_9968-model4.8 | -11.8785 |
|  | XP\_065064539\_9968-model4.9 | -11.8682 |
|  | XP\_065064539\_9968-model4.10 | -11.8622 |
|  | XP\_065064539\_9968-model4.11 | -11.788 |
|  | XP\_065064539\_9968-model4.12 | -11.7714 |
|  | XP\_065064539\_9968-model4.13 | -11.7635 |
|  | XP\_065064539\_9968-model4.14 | -11.7635 |
|  | XP\_065064539\_9968-model4.15 | -11.7365 |
|  | XP\_065064539\_9968-model4.16 | -11.7276 |
|  | XP\_065064539\_9968-model4.17 | -11.7196 |
|  | XP\_065064539\_9968-model4.18 | -11.7159 |
|  | XP\_065064539\_9968-model4.19 | -11.701 |
|  | XP\_065064539\_9968-model4.20 | -11.688 |
|  | XP\_065064539\_9968-model4.21 | -11.6649 |
|  | XP\_065064539\_9968-model4.22 | -11.6347 |
|  | XP\_065064539\_9968-model4.23 | -11.6264 |
|  | XP\_065064539\_9968-model4.24 | -11.6054 |
| # cluster 5 - Size: 5 |  |  |
|  | XP\_065064539\_9968-model5 | -11.9335 |
|  | XP\_065064539\_9968-model5.1 | -11.8935 |
|  | XP\_065064539\_9968-model5.2 | -11.8549 |
|  | XP\_065064539\_9968-model5.3 | -11.6999 |
|  | XP\_065064539\_9968-model5.4 | -11.6007 |
| # cluster 6 - Size: 9 |  |  |
|  | XP\_065064539\_9968-model6 | -11.8777 |
|  | XP\_065064539\_9968-model6.1 | -11.8121 |
|  | XP\_065064539\_9968-model6.2 | -11.8078 |
|  | XP\_065064539\_9968-model6.3 | -11.7675 |
|  | XP\_065064539\_9968-model6.4 | -11.7583 |
|  | XP\_065064539\_9968-model6.5 | -11.7313 |
|  | XP\_065064539\_9968-model6.6 | -11.6622 |
|  | XP\_065064539\_9968-model6.7 | -11.6441 |
|  | XP\_065064539\_9968-model6.8 | -11.4988 |
| # cluster 7 - Size: 2 |  |  |
|  | XP\_065064539\_9968-model7 | -11.7668 |
|  | XP\_065064539\_9968-model7.1 | -11.5978 |
| # cluster 8 - Size: 2 |  |  |
|  | XP\_065064539\_9968-model8 | -11.7445 |
|  | XP\_065064539\_9968-model8.1 | -11.7365 |
|  |  |  |
